# An Account of Diseases in an Eastern District of London, from October 20, to November 20, 1808

**Published:** 1808-12

**Authors:** 


### *An Account of Diseases in an Eastern District of London, from October 20, to November 20, 1808.*

| | |
|---|---|
| **ACUTE DISEASES.** | |
| Typhus - - - | 2 |
| Scarlatina Anginosa | 2 |
| Pleuritis - - | 1 |
| Rheumatismus Acutus | 3 |
| **CHRONIC DISEASES.** | |
| Tussis - - - | 7 |
| Tussis cum Dyspnœa | 6 |
| Phthisis Pulmonalis | 4 |
| Vomitus - - - | 3 |
| Diarrhœa - - - | 6 |
| Prolapsus Ani - - | 2 |
| Hemiplegia - - | 1 |
| Dyspepsia - - | 6 |
| Hypochondriasis - | 2 |
| Menorrhagia - - | 5 |
| Amenorrhœa - - | 6 |
| Fluor Albus - - | 7 |
| Rheumatismus Chronicus | 7 |
| **PUERPERAL DISEASES.** | |
| Peritonitis - - | 1 |
| Menorrhagia Lochialis | 5 |
| Mastodynia - - | 7 |
| Diarrhœa - - | 9 |
| **INFANTILE DISEASES.** | |
| Convulsio - - | 2 |
| Erysipelas Infantile | 3 |
| Ophthalmia - - | 3 |
| Vermes - - - | 2 |
| Diarrhœa - - | 5 |

Since the last Report an unusual quantity of rain has fallen, and very high winds have prevailed; and these have been frequently blowing from the north and north-east quarter. At intervals the sun has shone with considerable power, and in sheltered situations persons have found themselves comfortably warm, so that they have forgotten the danger to which a different situation might expose them. Various rheumatic affections have been the consequence of this inattention; and these, whether in the more acute or chronic form, have proved peculiarly troublesome and obstinate. Pains in the face and teeth have been very frequent. In some instances these have been mistaken for a common tooth-ache, and have been supposed to arise from one or more carious teeth, the removal of which, it was hoped, would prove the most effectual remedy. Teeth, however, have been drawn, but still the pain has continued, or has returned so soon as to convince the patient that he had mistaken the cause of his uneasiness, and that it was necessary to apply for some medical advice. Subsequent symptoms have discovered that rheumatic affection of the muscles of the face has been the principal cause of the uneasiness complained of, and a treatment adapted to the removal of such an affection has generally proved successful. Whilst a few grains of pulv. ipec. comp. have palliated the symptoms, or procured an intermission of pain; the use of the cortex has proved the most powerful means of lessening the frequency of the paroxysm, and of curing the disease.

